# ﻿Phylogeography of the Colombian water snake *Helicopsdanieli* Amaral, 1938 (Reptilia, Squamata, Dipsadidae) with comments on the systematics and evolution of the genus *Helicops* Wagler, 1828

**DOI:** 10.3897/zookeys.1215.128795

**Published:** 2024-10-17

**Authors:** Juan Pablo Hurtado-Gómez, Juan M. Daza, Mario Vargas-Ramírez, V. Deepak, Uwe Fritz

**Affiliations:** 1 Museum of Zoology, Senckenberg Natural History Collections Dresden, A.B. Meyer Building, 01109 Dresden, Germany Museum of Zoology Dresden Germany; 2 Grupo Herpetológico de Antioquia (GHA), Instituto de Biología, Universidad de Antioquia, Medellín, Colombia Universidad de Antioquia Medellín Colombia; 3 Grupo Biodiversidad y Conservación Genética, Instituto de Genética, Universidad Nacional de Colombia, Bogotá, Colombia Universidad Nacional de Colombia Bogotá Colombia; 4 Estación de Biología Tropical Roberto Franco (EBTRF), Universidad Nacional de Colombia, Villavicencio, Colombia Universidad Nacional de Colombia Villavicencio Colombia; 5 School of Natural and Environmental Sciences, Newcastle University, Newcastle upon Tyne NE1 7RU, UK Newcastle University Newcastle upon Tyne United Kingdom

**Keywords:** Actinoptergyii, Andalucia Pass, *Ichthyotachynectes* nom. nov., Myctophidae, Serpentes, subgenera, *Tachynectes*

## Abstract

The genus *Helicops* Wagler, 1828 comprises 20 species of semiaquatic snakes. It is mostly distributed in the cis-Andean region of South America, with only two trans-Andean species (*H.danieli*, *H.scalaris*). *Helicopsdanieli* is endemic to Colombia and occurs through most of the trans-Andean region. Herein two mitochondrial and two nuclear genomic markers were sequenced for 16 samples of *H.danieli* across most of its distribution range to understand its phylogeography. A dated tree was also generated with additional sequences from previous studies to infer the divergence times between *H.danieli* and its cis-Andean congeners and of lineages within *H.danieli*. Using previously published data, ancestral states were estimated for putative phenotypic synapomorphies for the major clades of *Helicops*. For *H.danieli*, four clades corresponding to the main river basins within its distribution were recovered. Our dated tree suggests that the ancestor of *H.danieli* diverged from its closest congeners in the late Miocene (8.7 Mya), which can be associated with the closure of the Andalucia Pass, south of the Eastern Cordillera. Divergence within *H.danieli* commenced 1.1 Mya. Within the genus *Helicops*, two distinct hemipenial morphologies were observed, which are suggested as putative synapomorphies for the two most basal clades. Recognition of these two clades as distinct subgenera, *Helicops* sensu stricto and *Tachynectes* Fitzinger, 1843 is proposed. For the junior homonym *Tachynectes* von der Mark, 1863, rarely applied to fossil fishes, the replacement name *Ichthyotachynectes***nom. nov.** is introduced. Furthermore, the evolution of another four phenotypic traits in *Helicops* and their phylogenetic utility are discussed.

## ﻿Introduction

The genus *Helicops* Wagler, 1828 currently contains 20 species, distributed through most of the South American subcontinent (Moraes-da-Silva et al. 2022; Schöneberg and Köhler 2022). Snakes in this genus are characterized by having semiaquatic habits, eyes and nostrils dorsally located, dorsal scales keeled, a single internasal scale, and an S-shaped sulcus spermaticus in the hemipenial lobes (Zaher 1999; Schöneberg and Köhler 2022). All *Helicops* species are distributed in cis-Andean South America, except for *H.scalaris* Jan, 1865, from the Lake Maracaibo Region, and *H.danieli* Amaral, 1937, which is confined to the trans-Andean lowlands of Colombia, the inter-Andean valley of the Magdalena River, the Caribbean floodplains, and the Pacific region (Rossman 2002a; Citeli et al. 2021).

*Helicopsdanieli* was originally described by Amaral (1937) based on a single specimen from the Río Carare, Santander, Colombia, on the eastern edge of the Magdalena Valley. Later, several authors extended the knowledge about the species’ morphology, describing the overall variation in scalation, measurements, and hemipenial morphology (Yuki 1994; Rossman 2002a; Citeli et al. 2021). Initially, Amaral (1937) suggested that *H.danieli* was related to *H.angulatus* and *H.scalaris*. Later, Nunes (2006) developed a morphology-based phylogenetic hypothesis and included *H.danieli* in a phylogenetic context for the first time. According to Nunes (2006), *H.danieli* is sister to a clade containing *H.angulatus*, *H.gomesi*, *H.pastazae*, *H.petersi*, *H.polylepis*, and *H.scalaris*. To date, none of the recent molecular-based phylogenies has included *H.danieli* (Costa et al. 2016; Moraes-da-Silva et al. 2019, 2021, 2022).

*Helicopsdanieli* is widely distributed across the trans-Andean lowlands of Colombia. This region comprises a variety of ecosystems and geographic units, including dry and moist forests (Etter et al. 2021), inter-Andean valleys, Pacific lowlands, the Caribbean plain, and several basins primarily associated with the Magdalena, Cauca, and Atrato rivers (Hernández-Camacho et al. 1992; Lynch et al. 1997; Mesa-S. et al. 2016). This suggests that phylogeographic variation exists in *H.danieli*.

In recent years, research on the systematics and taxonomy of the genus *Helicops* has expanded considerably, with six out of the twenty species described in the last two decades (da Frota 2005; Kawashita-Ribeiro et al. 2013; Costa et al. 2016; Moraes-da-Silva et al. 2019, 2021, 2022). Additionally, the number of taxa studied using molecular genetic approaches has increased, with up to 11 *Helicops* species analyzed to date (Moraes-da-Silva et al. 2021). Recent studies have mapped various phenotypic traits to identify synapomorphies, some of which (e.g., color pattern, subcaudal keels, reproductive mode; Moraes-da-Silva et al. 2022) have been useful for supporting minor clades within *Helicops*. However, no synapomorphies have been identified for the major clades of *Helicops*.

For the present study, we generated a dataset of four molecular markers to infer the phylogenetic position as well as the genetic and geographic structure of *H.danieli*. Additionally, we present a fossil-calibrated time tree for *Helicops* to estimate the divergence time for *H.danieli* and its cis-Andean congeners. Finally, we infer the ancestral states for five phenotypic characters of *Helicops* using our molecular phylogeny, discuss the evolution and phylogenetic value of these traits, and propose a subgeneric classification for *Helicops*.

## ﻿Materials and methods

### ﻿Sampling and laboratory procedures

**Figure 1.**
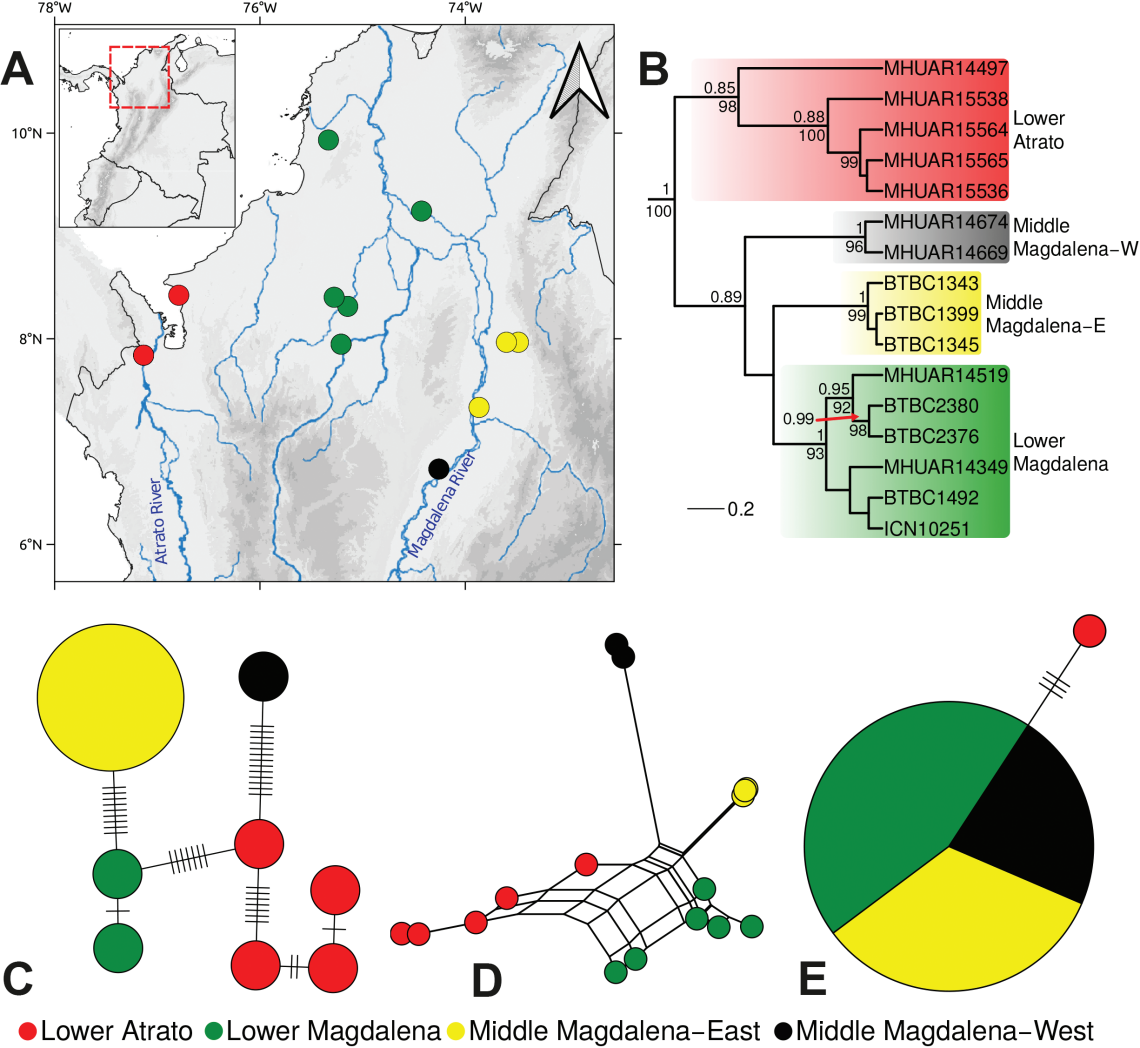
**A** Genetic sampling and clades for *Helicopsdanieli***B** Bayesian tree for *H.danieli* lineages (cropped from the complete tree, see Suppl. material 2: fig. S1) based on the concatenated alignment of three mitochondrial and four nuclear molecular markers (4624 bp); values above branches indicate Bayesian posterior probabilities (> 0.85), below branches are UltraFastBootstrap values (> 90) from the ML tree (Suppl. material 2: fig. S2) **C** haplotype network for *H.danieli* based on the concatenated mtDNA alignment of 16S and cyt *b* sequences **D** Neighbor-Net for *H.danieli* samples based on the same concatenated mtDNA alignment **E** haplotype network for *H.danieli* samples based on the nuclear Rag 1 fragment.

We used 16 samples of *H.danieli* from most of its range (Fig. 1A) and one of *H.pastazae* from tissues deposited in the Banco de Tejidos de la Biodiversidad, Instituto de Genética, Universidad Nacional de Colombia, Bogotá, Colombia (**BTBC**) and the Museo de Herpetología, Universidad de Antioquia, Medellín, Colombia (**MHUA**) (Suppl. material 1: table S1). DNA was extracted using the innuPREP DNA Micro Kit (Analytik Jena GmbH, Jena, Germany) following the manufacturer’s protocol. We obtained sequences from two mitochondrial (16S: 532 bp, cyt *b*: 770 bp) and two nuclear markers (C-mos: 566 bp, Rag1: 826 bp). PCR was conducted in a reaction volume of 25 µl, containing 20–40 ng of DNA, 1 unit *Taq* polymerase (Bioron GmbH, Ludwigshafen, Germany), the buffer recommended by the supplier (complete, 10×, containing MgCl_2_), 0.2 µM of each dNTP (Carl Roth GmbH + Co. KG, Karlsruhe, Germany), and 0.4 µM of each primer. Primers and PCR cycling conditions are described in the Suppl. material 1: table S2. Purification and sequencing followed Fritz et al. (2012). Sequences were edited with GENEIOUS 9.1.8 (Kearse et al. 2012) and aligned using MAFFT 7.39 (Katoh and Toh 2010) as implemented in GENEIOUS. To obtain a more robust hypothesis for the relationships within *Helicops*, we generated a concatenated alignment of 4624 bp length for the phylogenetic trees (see below). This alignment included three additional markers (mtDNA = 12S; nDNA = BDNF, NT3) available from previous studies (Moraes-da-Silva et al. 2019, 2021). Additional sequences for *Helicops* species and outgroups were downloaded from GenBank (Suppl. material 1: table S1). The sequences for 13 colubroid outgroup taxa originate from Zaher et al. (2019); the most distant species, the viperid *Bothropsatrox*, served for tree rooting. The individual alignments were concatenated using SEQUENCE MATRIX 1.8 (Vaidya et al. 2011). For the phylogenetic analyses, we included only one sample per species, except for *H.danieli*, for which we included all individuals, and for *H.angulatus*, which was recently retrieved as non-monophyletic (Murphy et al. 2020). For *H.angulatus*, we included one sample from Trinidad and another one from Brazil (Suppl. material 1: table S1). For the calculation of uncorrected *p* distances (see below), we included all available mitochondrial 16S and cyt *b* sequences.

### ﻿Phylogenetic analyses and time-calibrated tree

The best partition scheme and substitution models for analyzing the concatenated sequences were determined using MODELFINDER (Kalyaanamoorthy et al. 2017) as implemented in IQ-TREE 2.2 (Minh et al. 2020). Model selection was performed with the parameter ‘testmerge’ for model selection option (-m), which implements the ‘greedy’ algorithm of PARTITIONFINDER 2.0 (Lanfear et al. 2016) (Suppl. material 1: table S3).

Phylogenetic trees were constructed using Maximum Likelihood (**ML**) and Bayesian Inference (**BI**). The ML tree was calculated with IQ-TREE 2.2, using partitions and substitution models obtained with MODELFINDER (Suppl. material 1: table S3). Node support for the ML tree was assessed with 5000 Ultrafast Bootstrap replicates (**UFB**), considering branches with support values of 95% and above as highly supported (Minh et al. 2013). For the BI and the relaxed molecular clock calculations (see below), we used BEAST 2.7.5 (Bouckaert et al. 2019) and two independent chains of 50 million generations, sampling every 5000^th^ generation. For the BI analysis, we used the partitions obtained with MODELFINDER. However, substitution models were determined using BMODELTEST (Bouckaert and Drummond 2017) exploring all available models (Suppl. material 1: table S3). The Yule model was used for tree inference. For the time-calibrated tree, we applied the fast-normal relaxed clock model and three fossil calibration points from Zaher et al. (2019), as follows (fossil, minimum age, reference): (i) stem Colubroidea (Colubridae indet., 35.2 Mya; Smith 2013); (ii) stem Dipsadidae (*Paleoheterodontiheni*, 12.5 Mya; Holman 1964, 1977); and (iii) crown Natricidae (Natricidae incertae sedis, 13.8 Mya; Rage and Szyndlar 1986). Chain convergence and burn-in (20%) were examined using TRACER 1.7.1 (Rambaut et al. 2018). A maximum credibility tree was summarized with TREEANNOTATOR 2.7.5 implemented in BEAST 2.7.5 (Bouckaert et al. 2019). For tree annotation, plotting, and layout, we used the R program v. 4.3.1 (R Core Team 2023) in RSTUDIO (RStudio Team 2023) along with the packages ‘ape’ (Paradis and Schliep 2019), ‘phangorn’ (Schliep 2011), ‘phytools’ (Revell 2012), and the INKSCAPE software (https://www.inkscape.org).

### ﻿Haplotype networks, Neighbor-Nets, and genetic distances

Two parsimony networks were drawn for the *H.danieli* samples using the R package PEGAS 1.2 (Paradis 2010), one for the nuclear fragment Rag1 and the other for the concatenated mitochondrial alignment (16S and cyt *b*), acknowledging that mtDNA represents a single locus. Due to the low variation in the C-mos alignment, no network was calculated for this marker.

Phylogenetic networks for the concatenated mitochondrial alignment (16S and cyt *b*) were computed using the Neighbor-Net algorithm (Bryant and Moulton 2004), implemented in the R package ‘phangorn’ (Schliep 2011). Given that the algorithm does not run with alignments with approximately 50% missing data (JPHG pers. obs.), specimens with only the 16S marker in the mitochondrial alignment (the shortest one) were excluded from this analysis. Due to the limited variation in the concatenated nuclear alignment, no Neighbor-Net was drawn for it.

Finally, using MEGA 11 (Tamura et al. 2021) and the pairwise deletion option, uncorrected *p* distances were computed for the 16S and cyt *b* alignments for *Helicops* species and clades retrieved for *H.danieli* in the phylogenetic trees.

### ﻿Phenotypic data and ancestral state estimation

To identify putative phenotypic synapomorphies for the genus *Helicops*, we used the compiled data for all species from Murphy et al. (2020) and Moraes-da-Silva et al. (2022) on color pattern, keels on dorsal scales, subcaudal keels, and reproductive modes. Additionally, we incorporated all available information on hemipenial morphology from the literature, which included data for all *Helicops* species but *H.yacu*. The five phenotypic characters are: 1) dorsal color pattern, 2) strength of dorsal scale keels, 3) subcaudal keels, 4) reproductive mode, and 5) hemipenial lobe length.

To evaluate whether these phenotypic traits represent synapomorphies for the clades within *Helicops*, we performed an ancestral state estimation (**ASE**). First, we inferred the best fitting evolutionary model for each of the five characters, selecting among the three models equal rates (**ER**), symmetric rates (**SYM**), and all rates different (**ARD**) using the function ‘fitdiscrete’ in the R package ‘geiger’ (Harmon et al. 2007). The model with the lowest Akaike Information Criterion (AIC) value was chosen (Suppl. material 1: table S4).

For the ancestral state estimation, we used the function ‘ace’ in the R package ‘ape’. The Bayesian phylogenetic tree cropped to the genus *Helicops* served as input, along with the chosen model for each character. Since *H.angulatus* exhibits both oviparous and viviparous reproductive modes, we coded each of the two tips for the species with a different state.

## ﻿Results and discussion

**Figure 2.**
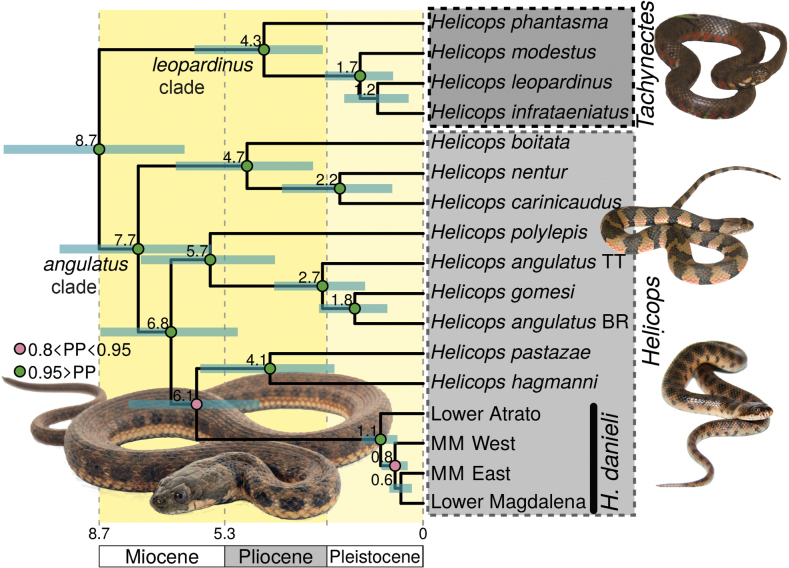
Bayesian time tree for the genus *Helicops* obtained with the concatenated alignment of three mtDNA and four nDNA markers, cropped from the complete tree (Suppl. material 2: fig. S1). *Helicopsdanieli* clades are collapsed (see Fig. 1B; Suppl. material 2: figs S1, S2). Values above branches are age estimates in million years; bluish green bars at nodes indicate 95% confidence intervals; circles at nodes represent Bayesian posterior probabilities (see legend). Boxes on branch tips and vertical names on the right indicate the subgeneric classification proposed in the present study. Abbreviations: BR, Brazil; MM, middle Magdalena; TT, Trinidad and Tobago. Photographs right: top, *H.leopardinus* (Corrientes, Argentina; A. Sabaliauskas, iNaturalist observation 94157056); center, *H.angulatus* (Guaviare, Colombia; J. P. Hurtado-Gómez); bottom, *H.pastazae* (Boyaca, Colombia; D. Gómez-Sánchez); photo left bottom, *H.danieli* (Los Katíos, Colombia; Juan M. Daza, MHUAR15536).

Our phylogenetic trees returned *Helicops* as maximally supported monophylum under both the ML and BI approaches (Fig. 2; Suppl. material 2: figs S1, S2). Within *Helicops*, two main clades were consistently recovered. The first clade, termed ‘*leopardinus* clade’, included *H.infrataeniatus*, *H.leopardinus*, *H.modestus*, and *H.phantasma* (Fig. 2). The second clade, the ‘*angulatus* clade’, comprised the remaining species: *H.angulatus*, *H.boitata*, *H.carinicaudus*, *H.danieli*, *H.gomesi*, *H.hagmanni*, *H.nentur*, *H.pastazae*, and *H.polylepis*. Notably, *H.pastazae*, which was studied for the first time using DNA sequence data, was found to be the sister taxon of *H.hagmanni* with high support (Fig. 2; Suppl. material 2: figs S1, S2).

*Helicopsdanieli* was recovered as monophyletic with high support by both tree-building approaches (Figs 1B, 2; Suppl. material 2: figs S1, S2) and as sister to the clade containing *H.hagmanni* and *H.pastazae* in a poorly supported clade (Fig. 2; Suppl. material 2: figs S1, S2). Within *H.danieli*, four main clades were revealed: one from the lower Atrato River, one from the lower Magdalena River, and two from the middle Magdalena River. In the middle Magdalena Basin (as defined by Mesa-S. et al. 2016), one clade (‘black’) occurs east and another one (‘yellow’) west of the river (Figs 1A, B, 2; Suppl. material 2: figs S1, S2). These clades were corroborated by the mitochondrial haplotype networks and Neighbor-Nets (Fig. 1C, D), though not by the nuclear Rag1 haplotype network (Fig. 1E). Our mitochondrial Neighbor-Net analysis indicated that the middle Magdalena groups (east and west) are well differentiated. In contrast, those from the lower Atrato (‘red’) and lower Magdalena (‘green’) show a high degree of interconnection, despite forming separate clusters (Fig. 1D). The relationships between clades varied significantly between analyses, but with relative higher node support in the BI tree (Figs 1B, 2; Suppl. material 2: figs S1, S3).

### ﻿Diversity and phylogeny of *Helicops*

The uncorrected *p* distances for the mitochondrial 16S fragment among *Helicops* species averaged 5.6%, ranging from 1.0% (between *H.leopardinus* and *H.infrataeniatus*) to 8.0% (between *H.gomesi* and *H.phantasma*; Table 1). For the cyt *b* marker, distances between *Helicops* species ranged from 9.7% (between *H.angulatus* and *H.pastazae*) to 14.3% (between *H.danieli* and *H.infrataeniatus*), averaging 11.7% (Table 1). Within *H.danieli*, uncorrected *p* distances for 16S and cyt *b* showed contrasting differentiation between the clades (Table 2). For the 16S marker, distances ranged from 0.1% (between the lower Magdalena and middle Magdalena east) to 2.0% (between the lower Atrato and middle Magdalena east). For the cyt *b* marker, distances ranged from 1.0% (between lower Atrato and lower Magdalena) to 2.0% (between lower Magdalena and middle Magdalena west).

**Table 1.** Means of interspecific uncorrected *p* distances (percentages) for 16S (below diagonal) and cyt *b* (above diagonal) sequences for *Helicops*. Sequence of taxa corresponds to Fig. 2. Cyt *b* sequences were only available for four taxa. BR, Brazil; TT, Trinidad and Tobago.

	*Helicops* species	1	2	3	4	5	6	7	8	9	10	11	12	13
1	*H.danieli*		-	11.4	12.9	-	-	13.4	-	-	-	-	14.3	-
2	*H.polylepis*	5.5		-	-	-	-	-	-	-	-	-	-	-
3	*H.angulatus* TT	5.7	4.5		7.6	-	-	9.7	-	-	-	-	11.1	-
4	*H.angulatus* BR	5.6	5.6	2.6		-	-	11.2	-	-	-	-	11.8	-
5	*H.gomesi*	5.8	5.4	1.5	2.3		-	-	-	-	-	-	-	-
6	*H.hagmanni*	6.4	5.4	4.7	5.1	4.8		-	-	-	-	-	-	-
7	*H.pastazae*	4.5	4.7	4.0	4.4	4.0	3.3		-	-	-	-	14.1	-
8	*H.boitata*	6.7	6.8	7.3	7.6	7.8	7.1	7.5		-	-	-	-	-
9	*H.carinicaudus*	6.6	5.6	6.3	6.0	6.8	6.8	5.9	5.4		-	-	-	-
10	*H.nentur*	6.9	4.9	6.8	6.4	7.3	5.9	6.3	4.2	2.3		-	-	-
11	*H.phantasma*	7.3	5.2	7.3	7.1	8.0	5.9	6.4	5.4	2.8	2.8		-	-
12	*H.infrataeniatus*	6.0	5.5	6.2	6.8	6.9	6.7	5.6	6.1	4.8	4.8	4.0		-
13	*H.leopardinus*	6.3	6.1	6.7	7.1	7.2	6.9	6.3	6.1	5.5	5.5	4.2	1.0	
14	*H.modestus*	5.9	5.3	6.1	6.9	6.9	6.8	6.0	6.1	5.4	5.3	4.5	1.2	1.6

**Table 2.** Means of uncorrected *p* distances (percentages) for 16S (below diagonal) and cyt *b* (above diagonal) sequences for *Helicopsdanieli* clades. MM, middle Magdalena.

*H.danieli* lineage		1	2	3	4
1	Lower Atrato	-	1.7	1.1	1.0
2	MM west	1.8	-	1.9	2.0
3	MM east	2.0	0.6	-	1.1
4	Lower Magdalena	1.7	0.1	0.4	-

Our dated phylogenetic tree suggests that diversification within *Helicops* commenced in the upper Miocene, approximately 8.7 Mya (Fig. 2; Suppl. material 2: fig. S1). The ‘*angulatus* clade’ started to diversify shortly after, around 7.7 Mya (upper Miocene). In contrast, the ‘*leopardinus* clade’ began to radiate more recently, in the early Pliocene, approximately 4.3 Mya. Speciation events continued throughout the Pliocene and Pleistocene. *Helicopsdanieli* diverged from its cis-Andean congeners at least 6.1 Mya during the upper Miocene. Differentiation within *H.danieli* commenced in the early Pleistocene, approximately 1.1 Mya.

**Figure 3.**
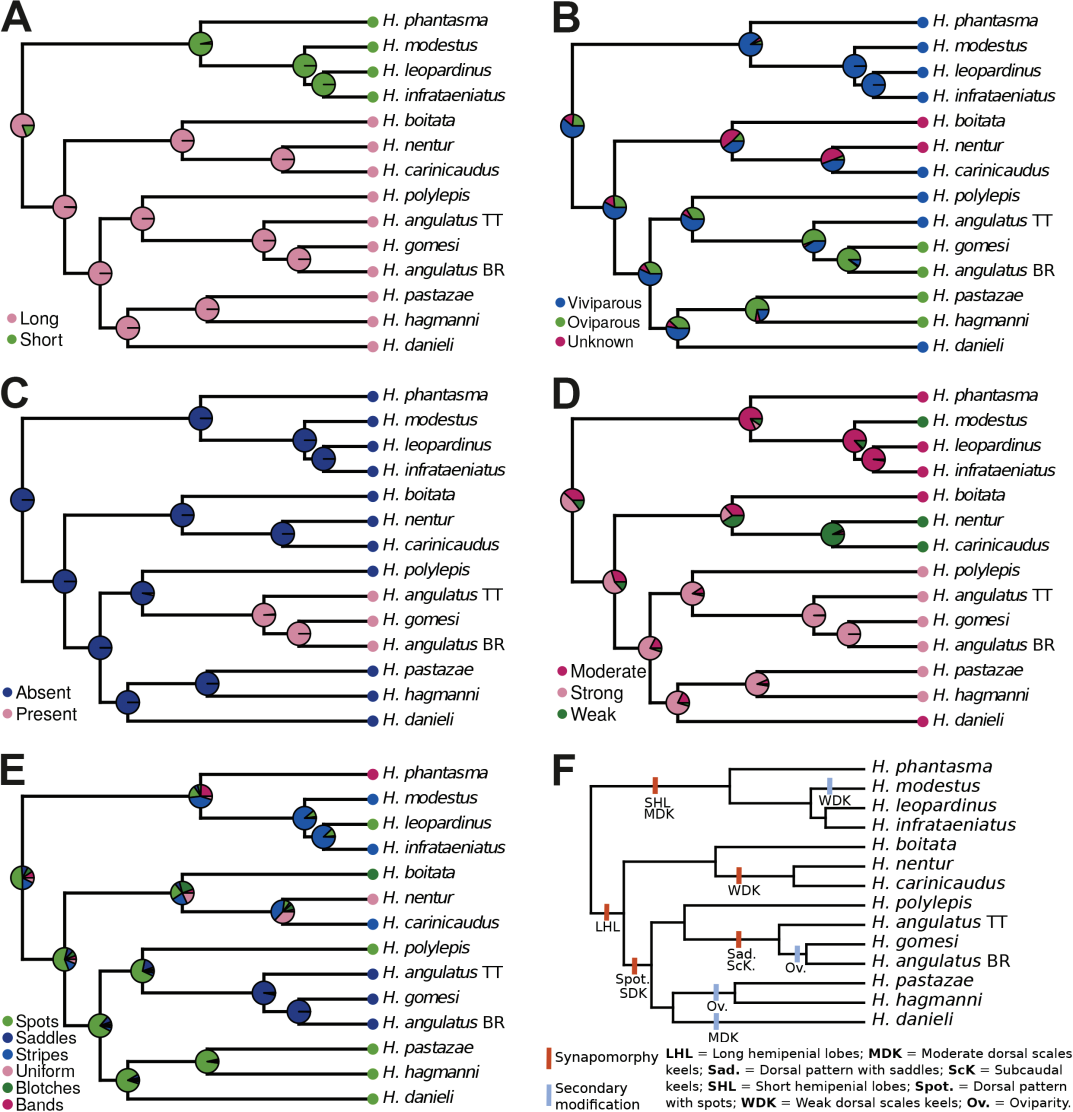
Ancestral state estimation using the summarized phylogeny for the genus *Helicops* for five phenotypic characters (**A** hemipenial lobe length **B** reproduction **C** subcaudal keels **D** strength of the dorsal scale keels **E** dorsal color pattern, and **F** summary of synapomorphies and secondary modifications for the nodes discussed in the text. Abbreviations (in bold) in **F** represent unambiguous synapomorphies. BR, Brazil; TT, Trinidad and Tobago.

**Figure 4.**
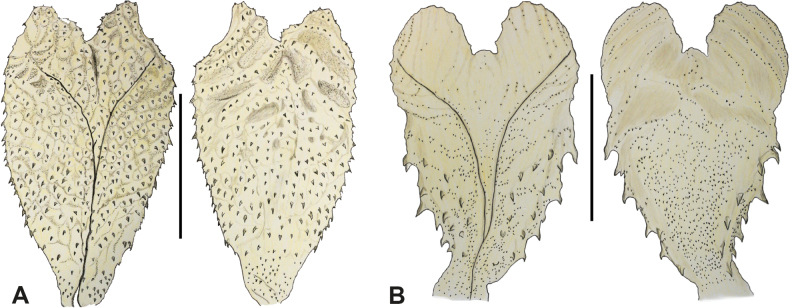
Hemipenes of **A***H.leopardinus* (based on UFMTR1504 from Moraes-da-Silva et al. 2021) and **B***H.danieli* (based on MHUAR15565) in sulcate (left) and asulcate (right) views. Scale bars: 10 mm. The hemipenis of *H.leopardinus* represents the morphology with short lobes characteristic for the subgenusTachynectes; the hemipenis of *H.danieli* represents the morphology with long lobes characteristic for the subgenusHelicops. Note that the hemipenis is homogeneously covered with spinules in (**A**), whereas large spines are confined to the hemipenial body (i.e., excluding the lobes) on the sulcate surface and the lateral regions in (**B**). Drawings: V. Deepak.

For all five phenotypic traits, the Equal Rates (ER) model was determined to be the best fit (Suppl. material 1: table S4). The ancestral state estimation (ASE) for the hemipenial lobe length indicates that each state represents a synapomorphy for the major clades within *Helicops* (Figs 3A, 4; Table 3). Short-lobed hemipenes (Fig. 4A) are prevalent among all species in the ‘*leopardinus* clade’ (which includes *H.infrataeniatus*, *H.leopardinus*, *H.modestus*, and *H.phantasma*). Conversely, long-lobed hemipenes (Fig. 4B) are characteristic of the species in the ‘*angulatus* clade’, including *H.angulatus*, *H.boitata*, *H.carinicaudus*, *H.danieli*, *H.gomesi*, *H.hagmanni*, *H.nentur*, *H.pastazae*, and *H.polylepis*.

**Table 3.** Classification of *Helicops* species as proposed in the present study and respective morphological character states.

Species	Subgenus	Dorsal pattern	Dorsal keel strength	Subcaudal keels	Reproductive mode	Hemipenial lobes	References
*H.acangussu* ^1^	*Helicops*	Spots	Moderate	Absent	Oviparous	Long	Moraes-da-Silva et al. (2022)
*H.angulatus*	*Helicops*	Saddles	Strong	Present	Bimodal	Long	Cope (1895), Zaher (1999), Nunes (2006)
*H.apiaka* ^1^	*Helicops*	Saddles	Strong	Present	Unknown	Long	Kawashita-Ribeiro et al. (2013)
*H.boitata*	*Helicops*	Blotches	Moderate	Absent	Unknown	Long	Moraes-da-Silva et al. (2019)
*H.carinicaudus*	*Helicops*	Stripes	Weak	Absent	Viviparous	Long	Zaher (1999), Nunes (2006)
*H.danieli*	*Helicops*	Spots	Moderate	Absent	Viviparous	Long	Zaher (1999), Nunes (2006)
*H.gomesi*	*Helicops*	Saddles	Strong	Present	Oviparous	Long	Nunes (2006)
*H.hagmanni*	*Helicops*	Spots	Strong	Absent	Oviparous	Long	Nunes (2006), Moraes-da-Silva et al. (2022)
*H.nentur*	*Helicops*	Uniform	Weak	Absent	Unknown	Long	Costa et al. (2016)
*H.pastazae*	*Helicops*	Spots	Strong	Absent	Oviparous	Long	Zaher (1999), Nunes (2006)
*H.petersi* ^1^	*Helicops*	Spots	Strong	Absent	Unknown	Long	Rossman (1976)
*H.polylepis*	*Helicops*	Spots	Strong	Absent	Viviparous	Long	Zaher (1999), Nunes (2006), Moraes-da-Silva et al. (2022)
*H.scalaris* ^1^	*Helicops*	Blotches	Strong	Present	Viviparous	Long	Nunes (2006)
*H.trivittatus* ^1^	*Helicops*	Stripes	Moderate	Absent	Viviparous	Long	Nunes (2006)
*H.yacu* ^1^	*Helicops*	Spots	Unknown	Unknown	Unknown	Unknown	Rossman and Dixon (1975), Rossman (1976)
*H.infrataeniatus*	*Tachynectes*	Stripes	Moderate	Absent	Viviparous	Short	Yuki and Lema (2005), Nunes (2006)
*H.leopardinus*	*Tachynectes*	Spots	Moderate	Absent	Viviparous	Short	Zaher (1999), Nunes (2006), Moraes-da-Silva et al. (2021)
*H.modestus*	*Tachynectes*	Stripes	Weak	Absent	Viviparous	Short	Zaher (1999), Nunes (2006)
*H.phantasma*	*Tachynectes*	Bands	Moderate	Absent	Viviparous	Short	Moraes-da-Silva et al. (2021)
*H.tapajonicus* ^1^	*Tachynectes*	Uniform	Weak	Absent	Unknown	Short	da Frota et al. (2005)

^1^ Species not sampled for molecular phylogenetics.

We additionally observed that hemipenes with short lobes have the organ body homogeneously covered with spinules, occasionally together with few enlarged spines (e.g., *H.phantasma*; Moraes-da-Silva et al. 2021) and body pockets immediately below the lobular crotch (Fig. 4A). On the other hand, hemipenes with long lobes have spinules and/or spines throughout the body (i.e., excluding the lobes), with the spines mainly located on the sulcate surface and the lateral regions of the hemipenial body; the lobes are ornamented with papillate calyces or flounces (Fig. 4B).

ASE for the reproductive mode suggests that viviparity is the most probable ancestral state for *Helicops* (Fig. 3B; Table 3). Viviparity is common among all sampled *Helicops* species (no data available for *H.boitata* and *H.nentur*), except for four species from two different clades: (i) *H.angulatus* and *H.gomesi* as well as (ii) *H.hagmanni* and *H.pastazae*. These four species are oviparous, with *H.angulatus* having both reproductive modes. Furthermore, ASE showed that subcaudal keels are present only in the clade containing *H.angulatus* and *H.gomesi*, representing an unambiguous synapomorphy of these two species (Fig. 3C; Table 3).

Each of the three character states regarding the strength of the dorsal scale keels corresponds to a synapomorphy for three clades within *Helicops* (Fig. 3D): i) for the clade containing *H.nentur* and *H.carinicaudus*, weak dorsal scale keels represent a synapomorphy; ii) for the ‘*leopardinus* clade’ (i.e., *H.infrataeniatus*, *H.leopardinus*, *H.modestus*, *H.phantasma*), moderately keeled dorsal scales are a synapomorphy, with a modification in *H.modestus*, which has weak keels; and iii) for the clade containing *H.angulatus*, *H.danieli*, *H.gomesi*, *H.hagmanni*, *H.pastazae*, and *H.polylepis*, strongly keeled dorsal scales are a synapomorphy, with a modification in *H.danieli* having moderately strong keels (Fig. 3D; Table 3).

ASE for the dorsal color pattern suggests that a spotted pattern is a synapomorphy for the clade comprised of *H.angulatus*, *H.danieli*, *H.gomesi*, *H.hagmanni*, *H.pastazae*, and *H.polylepis*. Within this group, a change occurs in the clade composed of *H.angulatus* and *H.gomesi*, for which a saddle pattern is an unambiguous synapomorphy (Fig. 3E; Table 3).

### ﻿Phylogeography of *Helicopsdanieli*

Our results retrieved *Helicopsdanieli* as monophyletic with a distinct geographic structure (Figs 1, 2; Suppl. material 2: figs S1, S2). We distinguish the four retrieved clades based on the Colombian freshwater ecoregions (Mesa-S. et al. 2016): lower Atrato, lower Magdalena, and two in the middle Magdalena, one on the eastern side and the other on the western side of the river course (Figs 1A, 2; Suppl. material 2: figs S1, S2). A similar differentiation pattern was observed in the pitviper *Bothropsasper*, where independent lineages were identified in the middle Magdalena, lower Magdalena, and in the Pacific lowlands (Saldarriaga Córdoba et al. 2017), with the latter partially corresponding to the lower Atrato clade of *H.danieli* (Figs 1A, B, 2; Suppl. material 2: figs S1, S2). Notably, the cladogenetic pattern found for *B.asper* by Saldarriaga Córdoba et al. (2017) resembles that of *H.danieli* in the Magdalena Basin, where the lower and middle Magdalena clades form a more inclusive clade. However, Saldarriaga Córdoba et al. (2017) did not find an east-west differentiation in *B.asper*. Given the recent diversification history of *H.danieli* (< 0.9 Mya), determining the causes of lineage differentiation is challenging due to the lack of detailed information on geological or climatic events in the distribution area during the relevant diversification period. Nonetheless, factors like isolation by distance or alterations in the river course have most likely influenced the genetic structuring of *H.danieli*. Further downstream, there is no clear east-west pattern in the Magdalena region. Records of the ‘yellow’ and ‘green’ clades are on both sides of the river, reflecting that the lower Magdalena is slow flowing and corresponds to a swamp system in the Momposina Depression.

However, for the middle Magdalena River, the phylogeography suggests an east-west differentiation of *H.danieli*, with the respective clades (‘black’ and ‘yellow’) being non-sister (Figs 1B, 2; Suppl. material 2: figs S1–S3). This pattern suggests that the Magdalena River acts as a significant barrier to gene flow. This is at first glance counterintuitive in the face of the semiaquatic habits of *H.danieli*. However, the species is typically found in ponds and small streams in shaded areas within associated vegetation (JPH-G, MV-R pers. obs.), like other *Helicops* species (Duellman 1978; Teixeira et al. 2017) and seems to avoid fast-flowing river sections like the middle Magdalena.

An east-west differentiation as in *H.danieli* has not been reported for other lowland reptiles in the middle Magdalena region (*Mabuya* spp., Pinto-Sánchez et al. 2015; *Podocnemislewyana*, Vargas-Ramírez et al. 2012; *Rhinoclemmysmelanosterna*, Vargas-Ramírez et al. 2013; *Caimancrocodilus*, Díaz-Moreno et al. 2021; *Bothropsasper*, Saldarriaga Córdoba et al. 2017) or other vertebrates (*Ateleshybridus*, Link et al. 2015) and, to our best knowledge, such a differentiation pattern is reported here for the first time for any vertebrate.

Moraes-da-Silva et al. (2022) previously suggested that the wide and fast flowing Madeira River acts as a barrier between *H.acangussu* and its closest relative *H.hagmanni* (based on morphology), as these species occur on opposite river sides. Even though this idea was not tested with a phylogenetic hypothesis (i.e., no molecular data available for *H.acangussu*), it supports that fast-flowing rivers are a barrier to gene flow in *Helicops*. Our study, therefore, provides the first detailed phylogeographic analysis of any *Helicops* species, offering direct evidence for the role of fast-flowing rivers as barriers to gene flow.

Genetic distances of the *H.danieli* clades ranged from 0.1 to 2.0% (16S) and from 1.0 to 2.0% (cyt *b*) (Table 2). For the 16S gene, the lineage from the lower Atrato (1.7–2.0%) showed the highest divergence from the others, while for cyt *b*, it was the lineage from the middle Magdalena west (1.7–2.0%). These distances are considerably lower than the mean distances observed between *Helicops* species (5.6% for 16S; 10.0% for cyt *b*) as well as interspecific distances found in other species within the dipsadid radiation (e.g., *Hydrodynastes*, Carvalho et al. 2020; *Caaeteboia*, Montingelli et al. 2020). This suggests that the four identified lineages of *H.danieli* represent early stages of genetic divergence that do not warrant taxonomic distinction.

We dated the split between the ancestor of *H.danieli* and its cis-Andean counterparts to the late Miocene, around 6.1 Mya (Fig. 2; Suppl. material 2: fig. S1). Fossil and geological evidence suggest that the trans-Andean and Amazon aquatic fauna were connected in Colombia until at least 5 Mya (Lundberg et al. 1998; Ballen and Moreno-Bernal 2019; Montes et al. 2021; Rodriguez-Muñoz et al. 2022) through a corridor south of the Eastern Cordillera called the Andalucia Pass. Given that most *Helicops* species and the whole Hydropsini radiation (also including the genera *Hydrops* and *Pseudoeryx*) are distributed in the cis-Andean region, it is likely that the ancestor of *H.danieli* migrated during the late Miocene from this region to the trans-Andean region, through this corridor, before the connection between the Magdalena and the Amazon Basins was interrupted by the upfold of the bridge between the Eastern and Central Cordilleras due to volcanic activity and fault propagation (Montes et al. 2021).

### ﻿Systematics of *Helicops*

#### ﻿A proposal for a subgeneric classification for *Helicops*

Our study provides the most comprehensive phylogenetic framework for the genus *Helicops* to date, covering 13 of the 20 currently recognized species (Moraes-da-Silva et al. 2022). Our phylogenetic trees (Fig. 2; Suppl. material 2: figs S1, S2) consistently recovered two more inclusive and highly supported clades within *Helicops*: the ‘*angulatus* clade’ and the ‘*leopardinus* clade’. These clades have also been consistently retrieved in previous molecular phylogenetic studies, even with different taxonomic sampling (Zaher et al. 2009, 2019; Grazziotin et al. 2012; Moraes-da-Silva et al. 2019, 2021). Notably, hemipenial morphology is diagnostic for each of the clades (Figs 3A, 4; Table 3). This morphological distinctiveness, combined with the strong phylogenetic support, allows the recognition of these two clades as subgenera of *Helicops* (Figs 2, 3; Table 3).

Based on this morphological and phylogenetic evidence, we propose assigning taxa in the ‘*angulatus* clade’ to the subgenusHelicops Wagler, 1828 sensu stricto (type species by monotypy: *Colubercarinicaudus* Wied-Neuwied, 1824 = *Helicopscarinicaudus*). Additionally, we propose placing the taxa in the ‘*leopardinus* clade’ in another subgenus for which the name *Tachynectes* Fitzinger, 1843 is available (type species by indication: *Homalopsisleopardina* Schlegel, 1837 = *Helicopsleopardinus*); see below under ‘Systematic account’.

#### ﻿Putative morphological synapomorphies for clades within *Helicops* and allocation of unsampled species

Beyond the hemipenial morphology that supports our proposed subgeneric classification, our ASE analysis for four additional phenotypic traits provides further phylogenetic information for various lineages within *Helicops* (Fig. 3; Table 3). These findings also allow us to tentatively assign unsampled species.

Our ASE analysis indicates that viviparity is the ancestral state for *Helicops*, with exceptions in two non-sister clades (Fig. 3B, F): one containing *H.hagmanni* and *H.pastazae*, and the other including *H.angulatus* and *H.gomesi* (with *H.angulatus* exhibiting both oviparity and viviparity; Table 3). According to our ASE analysis and phylogenetic hypothesis, oviparity has independently evolved twice within the subgenusHelicops (Fig. 3B; Table 3). Conversely, results of Moraes-da-Silva et al. (2022) suggest a single origin for oviparity, but this was because they inferred that all oviparous species (*H.angulatus*, *H.gomesi*, *H.hagmanni*, *H.pastazae*) are monophyletic.

The reproductive mode of six *Helicops* species remains unknown (Table 3), including two species in our phylogeny (*H.boitata* and *H.nentur*; Fig. 3B). Braz et al. (2016) proposed a geographic pattern for the bimodal reproductive mode in *H.angulatus*, but given that *H.angulatus* seems to be a species complex (cf. the deeply divergent non-sister samples of *H.angulatus* in our phylogeny; Fig. 2; Suppl. material 2: figs S1, S2), this bimodality might correspond rather to distinct taxa. Therefore, addressing these knowledge gaps requires a more comprehensive analysis for a definitive understanding of the evolution of reproductive modes in *Helicops*.

Moraes-da-Silva et al. (2022) proposed strong dorsal scale keels as a synapomorphy for the clade containing *H.angulatus*, *H.gomesi*, *H.hagmanni*, and *H.polylepis*. Our ASE results corroborate this hypothesis. Notably, *H.danieli* exhibits a modification with moderately strong dorsal keels. This putative synapomorphy is exclusive to this clade, with all other *Helicops* taxa having weak or moderate dorsal keels (Fig. 3D; Table 3).

Our ASE results also indicate that moderately keeled dorsal scales represent a synapomorphy for the subgenusTachynectes (referred to as the ‘*leopardinus* clade’ above), with a subsequent modification in *H.modestus* (with weak dorsal keels). Additionally, weak dorsal scale keels are identified as a synapomorphy for the clade containing *H.nentur* and *H.carinicaudus* (Fig. 3D, F). This is the first time that weak or moderate states of this character are identified as synapomorphies for clades within *Helicops*, highlighting the phylogenetic informativeness of dorsal scale carination.

Our results further suggest that the spotted dorsal pattern is also a synapomorphy for the clade containing *H.angulatus*, *H.danieli*, *H.gomesi*, *H.hagmanni*, *H.pastazae*, and *H.polylepis*, albeit with a secondary modification observed in the subclade *H.angulatus* + *H.gomesi*, which exhibits a dorsal pattern characterized by saddle-shaped blotches (Fig. 3E, F). The only other species in our study with a spotted pattern, *H.leopardinus* (subgenusTachynectes), likely represents a case of convergence, as no other closely related species shares this pattern.

For the subclade *H.angulatus* + *H.gomesi*, Moraes-da-Silva et al. (2022) previously suggested the dorsal color pattern consisting of saddle-shaped spots and subcaudal keels as putative synapomorphies, a hypothesis supported by our ASE results (Fig. 3E, F; Table 3). These traits are also shared by *H.apiaka* (Kawashita-Ribeiro et al. 2013), a species not included in our trees (Figs 2, 3; Suppl. material 2: figs S1, S2), which likely belongs to this clade (Table 3).

Subcaudal keels are also reported in *H.scalaris*, another unsampled species. While *H.scalaris* exhibits a polymorphic dorsal color pattern with individuals showing blotches (see photos in Rossman 2002b; Natera-Mumaw et al. 2015), some also display a saddle-like pattern (see iNaturalist observations: 129359317, 63932552, 19896494; Rossman 2002b). This suggests that *H.scalaris* might belong to the clade with saddle-shaped spots and subcaudal keels. However, *H.scalaris* is viviparous. If it truly belongs to this clade, it would support the idea of flexible reproductive modes within *Helicops* because *H.gomesi* is oviparous and *H.angulatus* exhibits both reproductive strategies. Regardless, both *H.apiaka* and *H.scalaris* possess hemipenes with long lobes, suggesting that they belong to the subgenusHelicops (Table 3).

Among the remaining species missing in our phylogenetic tree, *H.acangussu*Moraes-da-Silva et al. 2022, *H.petersi* Rossman, 1976, and *H.trivittatus* (Gray, 1849) possess hemipenes of the long-lobed morphotype and are therefore allocated to the subgenusHelicops (see Systematic account and Fig. 2; Table 3). *Helicopsacangussu* and *H.petersi* both exhibit a spotted dorsal pattern and have been associated with *H.hagmanni* and *H.pastazae*, respectively, primarily based on scale counts (Rossman 1976; Moraes-da-Silva et al. 2022). Thus, *H.acangussu* and *H.petersi* most likely belong to the clade formed by *H.hagmanni* and *H.pastazae*. This is further supported by the fact that *H.petersi* has strong dorsal scale keels, a putative synapomorphy of species in this clade, and *H.acangussu* is oviparous, a reproductive mode only occurring within the subgenusHelicops (Fig. 3; Table 3).

Another unsampled species is *H.yacu*, a taxon with a spotted dorsal pattern (Table 3), for which information regarding the hemipenial morphology is unavailable. Nevertheless, Rossman and Dixon (1975) and Rossman (1976) associate *H.yacu* with *H.polylepis*, *H.pastazae*, and *H.petersi*, based on scalation and color pattern. Later, Rossman and Abe (1979) suggested that *H.yacu* might be conspecific with *H.pastazae* (i.e., a subspecies). Therefore, based on the reported similarities in scutellation with the aforementioned species and its spotted dorsal pattern, we tentatively assign *H.yacu* to the subgenusHelicops.

The last species not included in our molecular phylogeny is *H.tapajonicus* da Frota, 2005. This species has hemipenes with short lobes and is therefore allocated to the subgenusTachynectes (see Fig. 2; Table 3; Systematic account).

### ﻿Comments on recent taxonomic changes regarding *Helicopsangulatus*

*Helicopscyclops* Cope, 1868 was recently resurrected from the synonymy of *H.angulatus* by Murphy et al. (2020) solely based on the morphology of the holotype (i.e., scale counts, color pattern, head shape). However, these traits show considerable overlap with *H.angulatus* (Murphy et al. 2020: table 2). Additionally, the locality of the holotype is imprecise (Bahia [State], Brazil), and Murphy et al. (2020) did not discuss the potential distribution range of *H.cyclops*. In the face of these limitations, we propose that *H.cyclops* should remain in the synonymy of *H.angulatus* pending additional evidence for its validity.

#### ﻿Diversification of *Helicops*

According to our results, the genus *Helicops* began to diversify in the late Miocene, around 9 Mya (Fig. 2; Suppl. material 2: fig. S1), aligning with the recent findings of Zaher et al. (2019), who suggested a similar time frame of 10.9 Mya. This period coincides with the existence of the Pebas System, a vast wetland that once covered most of the present-day Amazon Basin from approximately 20 to 5 Mya. The Pebas System was characterized by dynamic changes in landscape due to geological events and marine incursions throughout the Miocene (Hoorn et al. 2010, 2022). This dynamic wetland environment likely played a key role in the diversification of *Helicops*, and potentially the entire Hydropsini tribe (Suppl. material 2: fig. S1). This tribe includes semiaquatic snakes that diversified during the Miocene and are currently predominantly distributed in the Amazon Basin (Schöneberg and Köhler 2022).

### ﻿Systematic account

#### ﻿Phylum: Chordata

**Subphylum: Vertebrata**

**Superclass: Tetrapoda**

**Class: Reptilia**

**Order: Squamata**

**Family: Dipsadidae**

**Genus: *Helicops* Wagler, 1828**

##### ﻿Subgenus: Helicops

Taxon classificationAnimaliaSquamataDipsadidae

Wagler, 1828

CFDDE319-1AE1-5CE4-9433-FEF8D7670582

###### Type species.

*Colubercarinicaudus* Wied-Neuwied, 1824 (designated by Fitzinger 1843).

###### Diagnosis.

Members of the subgenusHelicops have long hemipenial lobes decorated with papillate flounces or calyces extending to the tips, but without spinules. The hemipenial body is covered both with spines and spinules; spines are concentrated on the sulcate surface and the lateral regions of the hemipenial body (Fig. 4B).

###### Content.

15 species, Helicops (Helicops) acangussuMoraes-da-Silva et al., 2022, H. (H.) angulatus (Linnaeus, 1758), H. (H.) apiaka Kawashita-Ribeiro, Ávila & Morais, 2013, H. (H.) boitataMoraes-da-Silva et al., 2019, H. (H.) carinicaudus (Wied-Neuwied, 1824), H. (H.) danieli Amaral, 1938, H. (H.) gomesi Amaral, 1922, H. (H.) hagmanni Roux, 1910, H. (H.) nenturCosta et al., 2016, H. (H.) pastazae Shreve, 1934, H. (H.) petersi Rossman, 1976, H. (H.) polylepis Günther, 1861, H. (H.) scalaris Jan, 1865, H. (H.) trivittatus (Gray, 1849), H. (H.) yacu Rossman & Dixon, 1975.

###### Comments.

Wagler (1828) coined the generic name *Helicops* to allocate *Colubercarinicaudus* (= *Helicopscarinicaudus*) and suggested that *Coluberangulatus* Linnaeus, 1758 (= *Helicopsangulatus*) and *Colubererytrogrammus* Palisot de Beauvois in Sonnini & Latreille, 1801 (= *Faranciaerytrogramma*) belonged to *Helicops* because of their similarity. Two years later, Wagler (1830) included in *Helicops* the following species: *H.carinicaudus*, *C.erytrogrammus*, *C.plicatilis*, *C.angulatus*, and *Natrixaspera* Wagler, 1824 (= *Helicopsangulatus*), without designation of a type species. Fitzinger (1843) designated *H.carinicaudus* as valid type species of *Helicops*. Since Wagler (1828) erected the name *Helicops*, the correct authorship has to be credited to Wagler (1828) and not Wagler (1830), as frequently seen (e.g., da Frota 2005; Kawashita-Ribeiro et al. 2013; Murphy et al. 2020; Schöneberg and Köhler 2022).

##### ﻿Subgenus: Tachynectes

Taxon classificationAnimaliaSquamataDipsadidae

Fitzinger, 1843

9389EAF2-ACA4-510F-8CF8-D7A44C8602D4

###### Type species.

*Homalopsisleopardina* Schlegel, 1837.

###### Diagnosis.

Members of the subgenusTachynectes have short hemipenial lobes decorated with spinules. The hemipenial body is homogeneously covered with spinules (Fig. 4A), occasionally a few enlarged spines may occur, e.g., in H. (T.) phantasma.

###### Content.

Five species, Helicops (Tachynectes) infrataeniatus Jan, 1865, H. (T.) leopardinus (Schlegel, 1837), H. (T.) modestus Günther, 1861, H. (T.) phantasmaMoraes-da-Silva et al., 2021, H. (T.) tapajonicus da Frota, 2005.

###### Comments.

*Tachynectes* von der Mark, 1863, erected for a genus of fossil fishes, is a primary junior homonym of *Tachynectes* Fitzinger, 1843. As *Tachynectes* von der Mark, 1863 has only been used four times in the past 50 years according to our searches (Google Scholar, Zoological Record: Sepkoski 2002; Albert et al. 2009; Dietze 2009; Schwarzhans and Carnevale 2021), it fails to meet the criterion in Article 23.9.2 of the International Code of Zoological Nomenclature (ICZN 1999) for prevailing usage and is therefore unavailable. As a replacement name for *Tachynectes* von der Mark, 1863, we propose *Ichthyotachynectes* nom. nov. to accommodate the fossil fish species previously assigned to *Tachynectes* von der Mark, 1863.

#### ﻿Phylum: Chordata

**Subphylum: Vertebrata**

**Superclass: Actinoptergyii**

**Class: Teleostei**

**Order: Myctophiformes**

**Family: Myctophidae**

##### ﻿Genus: Ichthyotachynectes nom. nov.

Taxon classificationAnimaliaMyctophiformesMyctophidae

18DBCC86-8DF5-5BE1-9FD8-83C3BF83FA70

https://zoobank.org/3B4D09B3-9E5A-4F4F-82FF-CBCE571D0533

###### Synonymy.

*Tachynectes* von der Mark, 1863 (invalid junior homonym of *Tachynectes* Fitzinger, 1843)

###### Type species.

*Tachynectesmacrodactylus* von der Marck, 1863.

###### Diagnosis.

For diagnosis and synapomorphies, see von der Marck (1863) and Dietze (2009).

###### Content.

Three species according to Dietze (2009), *Ichthyotachynectesmacrodactylus* (von der Marck, 1863), comb. nov., *I.longipes* (von der Marck, 1863), comb. nov., *I.brachypterygius* (von der Marck, 1863), comb. nov.

###### Etymology.

The new name (male gender) means “fish that swims fast” (from the classic Greek ichthyos = fish, tachys = fast, nectes = swimming). The name intends to keep the initial meaning of *Tachynectes* (fast swimmer), but adding a prefix indicating the taxonomic group.

###### Comments.

The homonymy of *Tachynectes* Fitzinger, 1843 and *Tachynectes* von der Mark, 1863 was already acknowledged by White and Moy-Thomas (1941), but overlooked by subsequent authors.

## ﻿Conclusions

Our study reveals a pronounced phylogeographic pattern in *H.danieli*, with four distinct lineages (Figs 1, 2; Suppl. material 2: figs S1, S2). Interestingly, these lineages exhibit clear differentiation on the eastern and western sides of the middle Magdalena River, suggesting this river acts as a barrier to gene flow for this aquatic snake species. Although our sampling did not extend to the southwestern Pacific region of Colombia, it is plausible that populations there are sister to the lower Atrato lineage, mirroring patterns observed in other taxa (e.g., *Mabuya* spp., Pinto-Sánchez et al. 2015; *Bothropsasper*, Saldarriaga Córdoba et al. 2017).

Moreover, we propose a subgeneric classification for *Helicops* based on the molecular phylogeny and hemipenial morphology with two subgenera: *Helicops* and *Tachynectes*. Additionally, we offer a new interpretation of four further phenotypic and natural history traits (i.e., reproductive mode, dorsal scale keel strength, subcaudal keels, dorsal color pattern) and their value as putative synapomorphies for lineages within the subgenusHelicops. This reinterpretation allows us to propose the most plausible phylogenetic placement for the seven *Helicops* species not included in our molecular phylogeny.

## Supplementary Material

XML Treatment for Helicops

XML Treatment for Tachynectes

XML Treatment for Ichthyotachynectes

## ﻿Additional information

### Author ORCIDs

Juan Pablo Hurtado Gómez https://orcid.org/0000-0003-4351-2834

Mario Vargas-Ramírez https://orcid.org/0000-0001-8974-3430

V. Deepak https://orcid.org/0000-0002-8826-9367

Uwe Fritz https://orcid.org/0000-0002-6740-7214

### Data availability

All of the data that support the findings of this study are available in the main text or Supplementary Information.

## Supplementary materials

Supplementary material 1 Supplementary tables Data type xlsx Explanation note **table S1.** Samples and their respective GenBank/ENA accession numbers and (only for the newly generated sequences) localities for the individuals used in the molecular analyses. **table S2.** Primers, sources, and PCR conditions for each of the four molecular markers. **table S3.** Partitions obtained with MODELFINDER as implemented in IQ-TREE for the concatenated analysis of six molecular markers (mtDNA = 12S, 16S, cyt *b*; nDNA = BDNF, C-mos, NT3, Rag1). **table S4.** Akaike Information Criterion values obtained for each of the five morphological characters and the three tested models. https://binary.pensoft.net/file/1155743This dataset is made available under the Open Database License (http://opendatacommons.org/licenses/odbl/1.0/). The Open Database License (ODbL) is a license agreement intended to allow users to freely share, modify, and use this Dataset while maintaining this same freedom for others, provided that the original source and author(s) are credited.Juan Pablo Hurtado-Gómez, Juan M. Daza, Mario Vargas-Ramírez, V. Deepak, Uwe Fritz

Supplementary material 2 Supplementary figures Data type pdf Explanation note **fig. S1.** Complete Bayesian dated tree obtained with the concatenated alignment of three mtDNA and four nDNA molecular markers. BR, Brazil; TT, Trinidad and Tobago. **fig. S2.** Complete Maximum Likelihood tree obtained with the concatenated alignment of three mtDNA and four nDNA markers. Values above branches indicate UltraFast bootstrap support. BR, Brazil; TT, Trinidad and Tobago. **fig. S3.** Detailed comparison between the Bayesian (left) and Maximum Likelihood (right) topologies obtained for *Helicopsdanieli*, cropped from Suppl. material 2: figs S1, S2, respectively. Values above branches on the left tree indicate Bayesian posterior probabilities, and on the right tree, UltraFast bootstrap support. https://binary.pensoft.net/file/1155737This dataset is made available under the Open Database License (http://opendatacommons.org/licenses/odbl/1.0/). The Open Database License (ODbL) is a license agreement intended to allow users to freely share, modify, and use this Dataset while maintaining this same freedom for others, provided that the original source and author(s) are credited.Juan Pablo Hurtado-Gómez, Juan M. Daza, Mario Vargas-Ramírez, V. Deepak, Uwe Fritz
