# Supplementary figures and images for: ﻿Phylogeography of the Colombian water snake Helicopsdanieli Amaral, 1938 (Reptilia, Squamata, Dipsadidae) with comments on the systematics and evolution of the genus Helicops Wagler, 1828
Source: Zookeys. 2024 Oct 17;1215:335–58. doi: 10.3897/zookeys.1215.128795 (PMC11502946; doi:10.3897/zookeys.1215.128795)

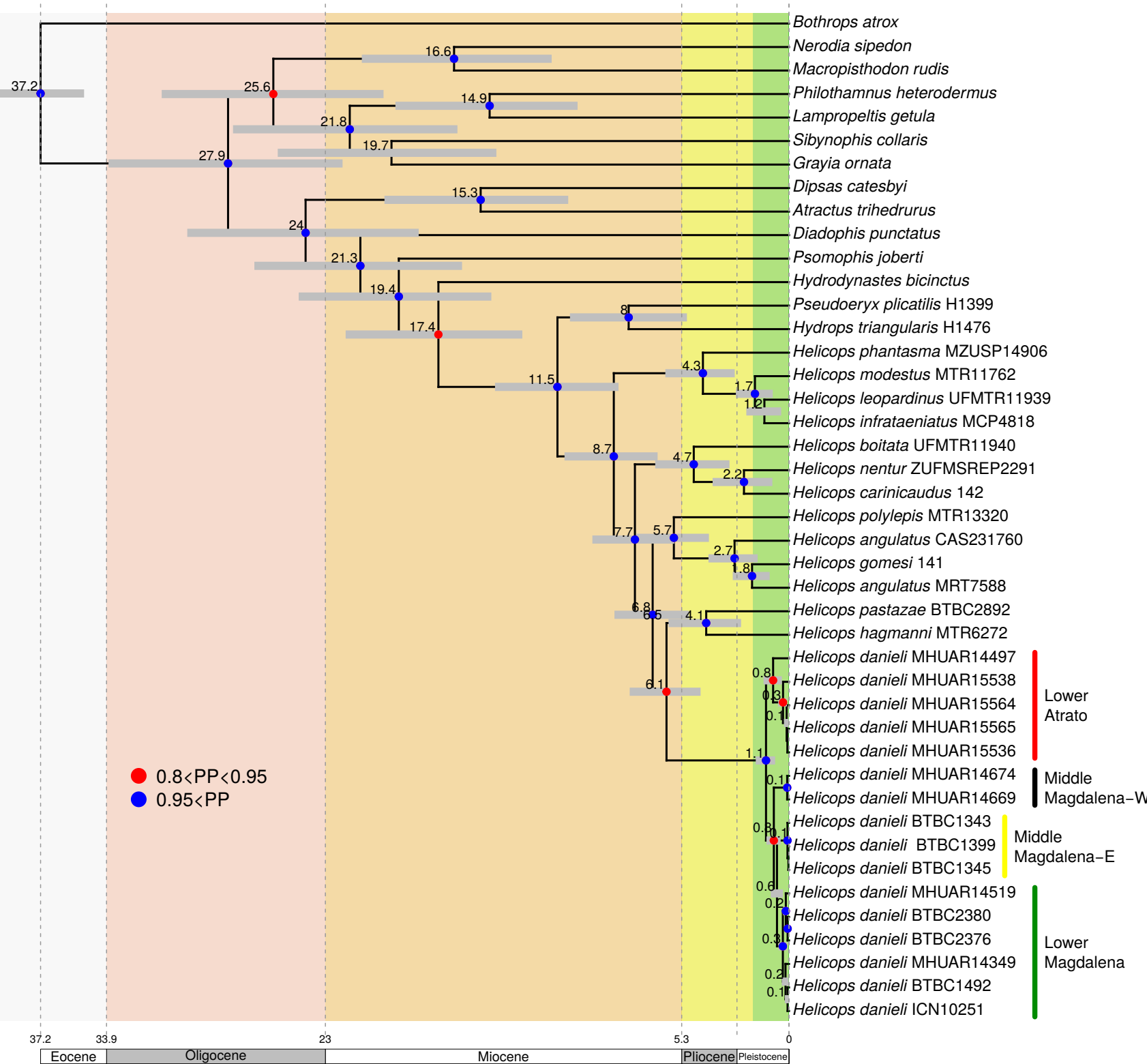

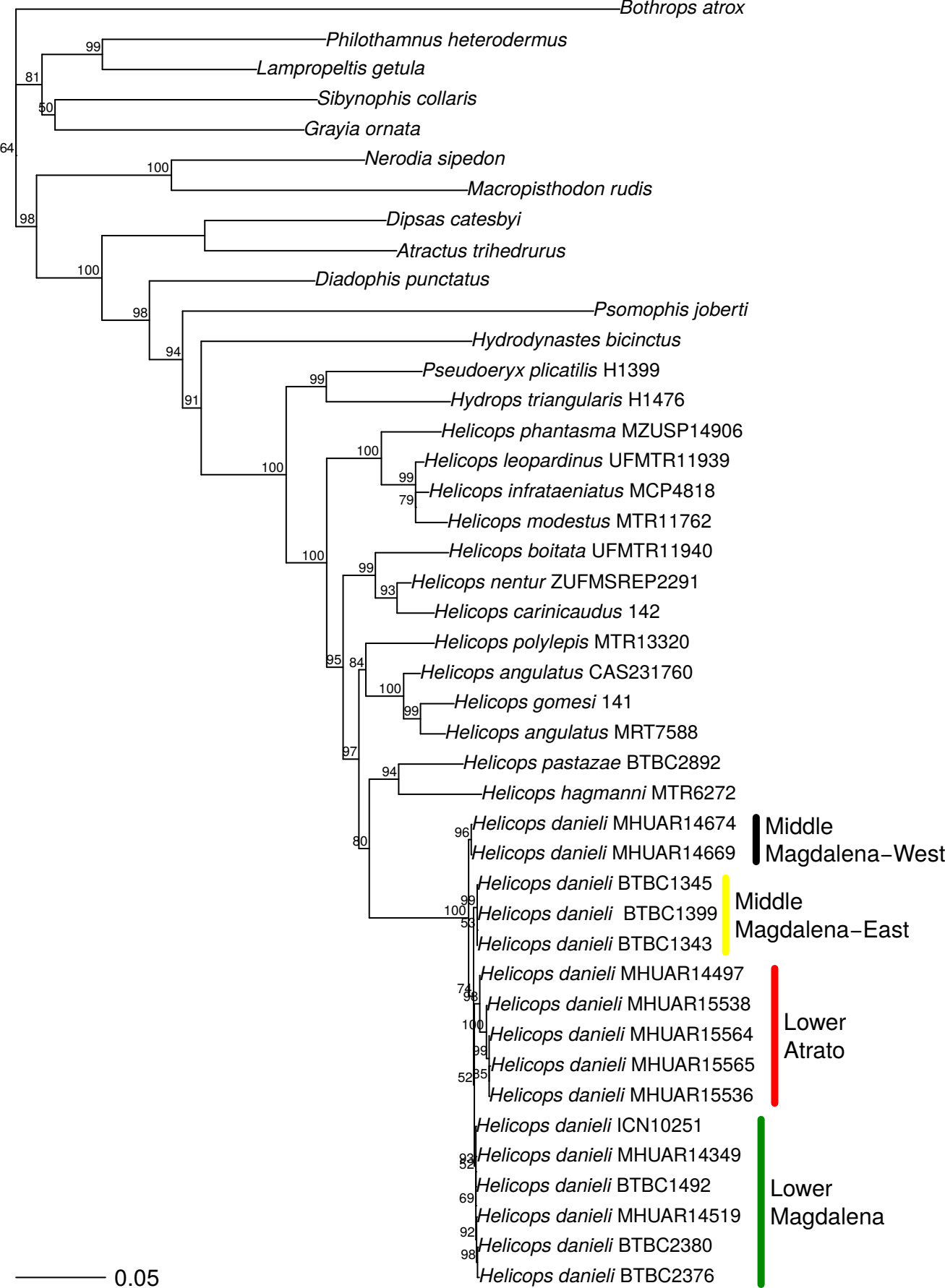

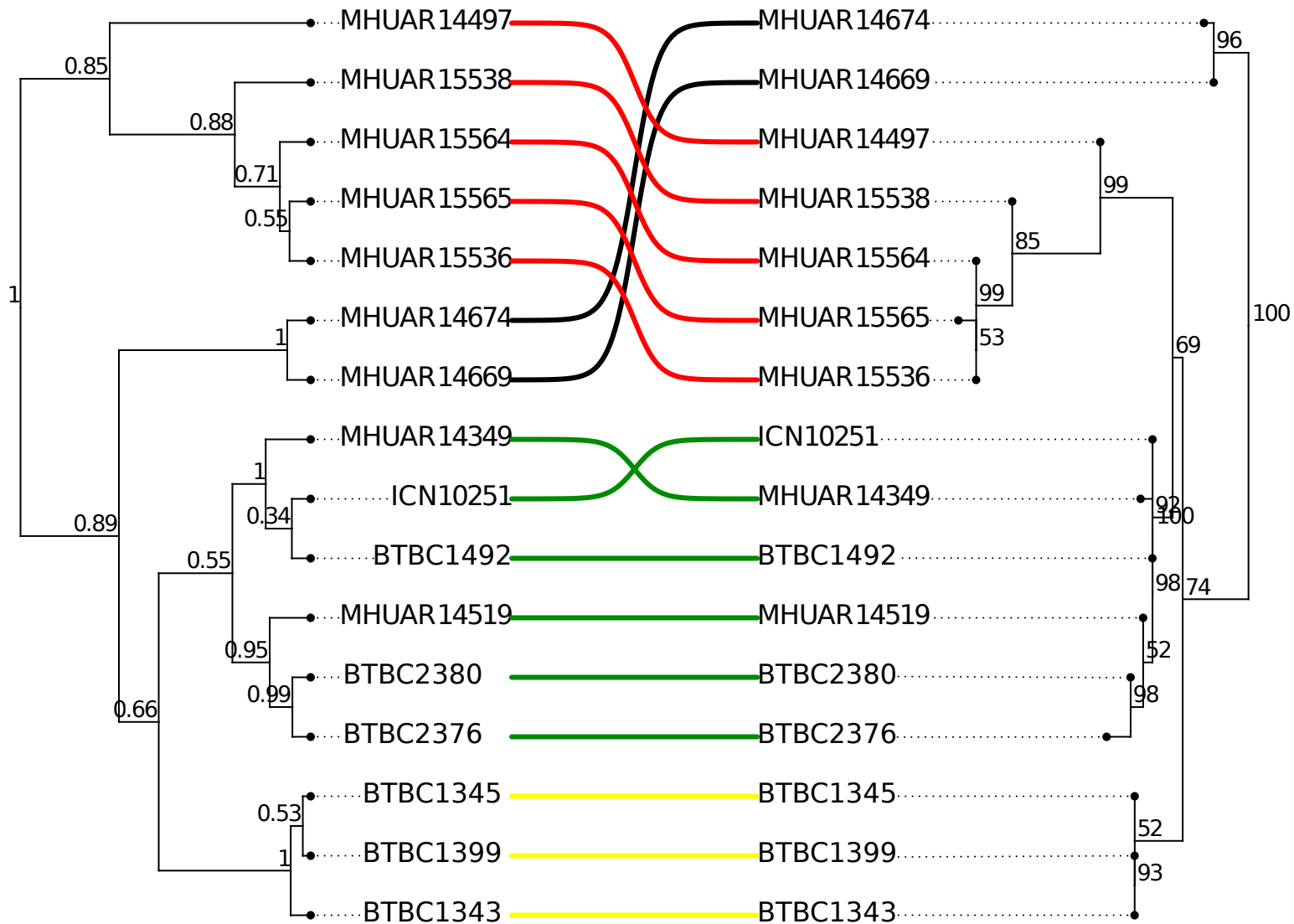

Supplement: Supplementary material 2 — Supplementary figures [file zookeys-1215-335_article-128795__-s002.pdf]
